# A rolling bearing fault diagnosis method based on an improved parallel one-dimensional convolutional neural network

**DOI:** 10.1371/journal.pone.0327206

**Published:** 2025-08-11

**Authors:** Hongwei Bai, Weiyan Tong, Zhenkun Geng, Cheng Gao

**Affiliations:** School of Chemical Process Automation, Shenyang University of Technology, Liaoyang, China; Newcastle University, UNITED KINGDOM OF GREAT BRITAIN AND NORTHERN IRELAND

## Abstract

As a critical component of industrial equipment, the fault diagnosis of rolling bearings is essential for reducing unplanned downtime and improving equipment reliability. Existing methods achieve an accuracy of no more than 92% in low signal-to-noise ratio environments. To address this issue, this paper proposes an improved parallel one-dimensional convolutional neural network model, which integrates a parallel dual-channel convolutional kernel, a gated recurrent unit, and an attention mechanism. The classification is performed using a global max-pooling layer followed by a Softmax layer. This dual-channel configuration captures both global and local features, decreases parameter redundancy, and reduces overfitting risk. Meanwhile, the GRU addresses the vanishing gradient issue and models long-term dependencies. Additionally, the attention mechanism emphasizes crucial features dynamically, improving feature selection and generalization. The global max-pooling layer replaces the fully connected layer, reducing the number of parameters, improving computational efficiency, and lowering the risk of overfitting. Experimental results demonstrate that the proposed model achieves superior performance in fault diagnosis, attaining an accuracy of 99.62%, significantly outperforming traditional CNNs and other benchmark methods.

## 1 Introduction

Liang et al. [1] highlighted that rolling bearings, as critical components widely used in various mechanical systems, are prone to failure due to prolonged operation in complex environments, thereby posing a significant threat to equipment safety and reliability. Wang et al. [2] emphasized that fault diagnosis of rolling bearings has become an essential approach for ensuring stable operation, reducing maintenance costs, and improving production efficiency. Condition monitoring through fault diagnosis enables early fault detection and prediction of its progression. However. Sun et al. [3] pointed out that the complex structure and harsh operating conditions of rolling bearings complicate fault feature extraction, as noise and operational signals often obscure the relevant features. Therefore, developing efficient fault diagnosis methods is of great significance.

Rui et al. [4] reported that deep learning techniques have made remarkable progress in the field of intelligent diagnosis in recent years. Chen et al. [5] demonstrated that the automatic feature extraction capability of deep learning effectively compensates for the limitations of traditional methods in recognizing complex fault patterns, thereby making the diagnosis process more intelligent and efficient. Krizhevsky et al. [6] identified convolutional neural networks (CNNs) as a typical deep learning method capable of enabling end-to-end learning. LeCun et al. [7] further demonstrated that CNNs can directly extract diagnostic results from raw data, thereby improving system efficiency and maintainability. Yu et al. [8] stated that with advancements in computational resources, the proliferation of big data, and continuous algorithm optimization, deep learning techniques have achieved significant success across various fields.

Deep Belief Networks (DBNs) typically consist of multiple restricted Boltzmann machines (RBMs) and a classifier at the top layer. Through a multi-layer structure, DBNs learn hierarchical representations of data, effectively capturing complex patterns. Shao et al. [9] proposed an improved DBN-based fault diagnosis model that uses pre-training with an energy function followed by fine-tuning with stochastic gradient descent, significantly enhancing classification accuracy. Tang et al. [10] introduced a frequency-domain-based DBN model and validated its effectiveness using datasets from automotive gearboxes and train bearings, demonstrating high recognition rates. Despite the widespread application of deep belief networks (DBNs), Jayadharshini et al. [11] noted that they exhibit certain limitations compared to CNNs when handling specific data types. DBNs heavily depend on input data structure and struggle with capturing complex spatial or temporal relationships. Yemi et al. [12] highlighted that CNNs excel at extracting local features, especially for image and time-series data, by leveraging convolutional layers to capture spatial and temporal dependencies. As a result, CNNs are widely applied in image classification, object detection, and fault diagnosis. Their end-to-end learning capability allows direct feature extraction and classification from raw data, providing robust support for rolling bearing fault diagnosis. Jiang et al. [13] pointed out that CNNs face challenges in robustness under noisy conditions, particularly in low signal-to-noise ratio (SNR) environments, where accurate fault type identification becomes difficult. To address these issues, researchers have explored hybrid models that integrate CNNs with other architectures to enhance performance. Alameh et al. [14] noted that recurrent neural networks (RNNs) are well-suited for sequential data processing by leveraging historical information to analyze current inputs. They have been extensively applied in time-series analysis and fault diagnosis.

An attention-based dual-selection RNN model that mitigates gradient explosion issues and achieved promising results in photovoltaic power prediction. Gargees et al. [15] integrated deep feature clustering with RNNs to classify using unlabeled data, yielding satisfactory outcomes.

As a classical deep learning model, CNNs have demonstrated exceptional performance across various domains, particularly in image and signal processing. CNNs can also be applied to textual data, capturing local correlations to learn key information from text. Ma et al. [16] developed a one-dimensional densely connected CNN model that improved diagnostic accuracy for wind turbine gearboxes. Zhang et al. [17] designed a deep CNN model with wide and narrow convolutional kernels to enhance bearing fault diagnosis accuracy. Eren et al. [18] proposed a one-dimensional CNN-based model that effectively integrates feature extraction and classification. Yao et al. [19] introduced a parallel one-dimensional CNN model that extracts frequency- and time-domain features of vibration signals, leading to improved diagnostic performance. Zhou et al. [20] incorporated gated recurrent units (GRUs) into deep CNNs to address gradient explosion issues and enhance noise robustness. Zhang et al. [21] applied deep one-dimensional CNNs with dual-channel information fusion for fire detection, achieving favorable results.

Although deep learning models can handle high-dimensional data and automatically extract features, several challenges remain. These models require large amounts of labeled data for training, which can be costly in industrial applications. Moreover, deep learning models involve complex training processes and substantial computational resources, particularly when dealing with high-dimensional data, which may hinder real-time monitoring applications. Additionally, deep learning models exhibit poor robustness to noise and anomalies, especially in low-SNR environments, where diagnostic accuracy may be compromised. Enhancing model robustness and adaptability under varying working conditions remains a key research focus.

Recent studies have increasingly focused on enhancing the robustness of deep learning models for fault diagnosis under challenging conditions, such as small sample sizes, strong noise interference, and varying operating environments. Qiu et al. [22] developed an enhanced residual shrinkage network that significantly improves fault identification performance in complex noisy backgrounds. Liu et al. [23] proposed a method that integrates Gramian angular difference fields with a dynamically self-calibrated convolutional module to boost time-series feature extraction. Xu et al. [24] introduced a multi-branch convolutional architecture designed to capture diverse feature scales for improved diagnostic accuracy. Yu et al. [25] addressed the issue of data scarcity by combining continuous wavelet transform with a multi-scale kernel attention mechanism in the CWMS-GAN framework. Similarly, Wu et al. [26] utilized an auxiliary classifier generative adversarial network to improve fault classification performance under limited data conditions.

In addition, Lu et al. [27] implemented a one-dimensional convolutional neural network to enable effective state perception of conveyor systems in complex industrial environments. Zhang et al. [28] integrated graph convolutional networks into a CNN-based model for capturing structural dependencies in fault data. Yan et al. [29] proposed a lightweight CNN tailored for real-time fault diagnosis on edge computing devices, offering a practical solution for deployment in resource-constrained scenarios.

These recent advances indicate a clear trend toward the development of diagnostic frameworks that are robust, generalizable, and suitable for industrial implementation. Nevertheless, existing approaches still face several limitations, including high model complexity, sensitivity to extremely low signal-to-noise ratios, and insufficient integration of time- and frequency-domain information. To address these challenges, this study proposes an improved model that combines a dual-channel convolutional structure with gated recurrent units (GRU) and attention mechanisms, aiming to enhance diagnostic performance and interpretability in complex working environments.

Building upon prior studies such as Fang et al. [30], who integrated time-frequency analysis for effective denoising and feature extraction to enhance the diagnostic accuracy and reliability of rolling bearings, this paper proposes an improved parallel one-dimensional convolutional neural network (CNN)-based fault diagnosis method. The proposed framework further advances diagnostic robustness under strong noise and varying operating conditions by incorporating gated recurrent units (GRU) and a convolutional block attention module (CBAM).

In this study, we propose a parallel one-dimensional convolutional neural network (P1DCNN) framework that integrates gated recurrent units (GRU) and a convolutional block attention module (CBAM) for robust fault diagnosis of rolling bearings under strong noise and variable operating conditions.

The main contributions of this study are summarized as follows:

(1) A parallel dual-channel convolutional framework is proposed to extract features independently from the time and frequency domains of raw vibration signals. This design enhances the model’s expressive capacity and enables the effective fusion of complementary information, which is often suppressed in single-domain feature extraction under noisy conditions.
(2) A gated recurrent unit (GRU) module is incorporated to model temporal dependencies and non-stationary characteristics in fault signals. Compared to conventional CNN and DBN-based approaches, this module more accurately captures the dynamic degradation behavior of rotating machinery, especially in scenarios involving long-term monitoring.
(3) A lightweight channel-spatial attention mechanism is introduced to selectively amplify salient features while suppressing irrelevant noise. This enhancement improves the model’s robustness under low signal-to-noise ratio (SNR) conditions without introducing significant computational overhead.
(4) Comprehensive experiments conducted on benchmark datasets and simulated industrial environments demonstrate that the proposed method consistently outperforms several representative models—including standard CNNs, CNN-GRU hybrids, and GAN-based classifiers—in terms of diagnostic accuracy, generalization ability, and noise tolerance, confirming its practical applicability for real-world fault monitoring tasks.

The remainder of this study is organized as follows. Section 2 presents the proposed model architecture. Section 3 details the experimental settings and results. Finally, Section 4 concludes the work and discusses future directions.

## 2 Fundamental principles

### 2.1 Convolutional neural networks

Due to the temporal nature of rolling bearing vibration signals, one-dimensional convolutional neural networks (1D-CNNs) are effective in capturing key time-domain features while reducing computational cost. Mirela et al. [31] noted that CNNs are capable of processing various data types-including time-series, images, and videos—making them suitable for vibration signal analysis.

Traditional methods such as time-domain statistics, STFT, and WT depend on manual feature design, limiting adaptability. In contrast, CNNs can automatically learn discriminative features through deep architectures. Guo et al. [32] demonstrated that CNNs reduce manual intervention and outperform fully connected networks by leveraging local receptive fields and weight sharing to lower complexity.

(1) Convolutional Layer

**Fig 1:**
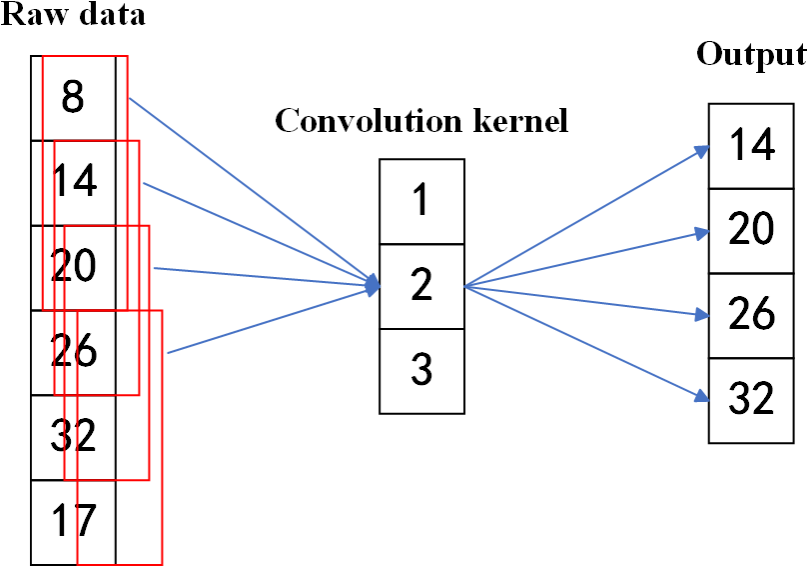
Diagram of one-dimensional data convolution operation. The convolution kernel slides over the input data and performs weighted summation at each position to generate the output.

Xu et al. [33] emphasized that convolutional layers extract local patterns through shared-weight kernels using a sliding window, enabling progressive abstraction with fewer parameters. Zhang et al. [34] further noted that this hierarchical representation enhances computational efficiency and feature expressiveness. The 1D convolution process is illustrated in Fig 1.

In a convolutional neural network, convolution operations are performed on the input data using convolutional kernels, as shown in Equation (1):


$$\begin{array}{c} x_{i+1}=W_{i}⊗x_{i}+b_{i} \end{array}$$ (1)

In this equations,

$x_{i}$ represents the input features of the i-th layer,

$W_{i}$ denotes the convolution kernel weights of that layer,

⊗ represents the convolution operation,

$b_{i}$ is the bias term,

$x_{i}+1$ is the output feature map after convolution.

**Fig 2:**
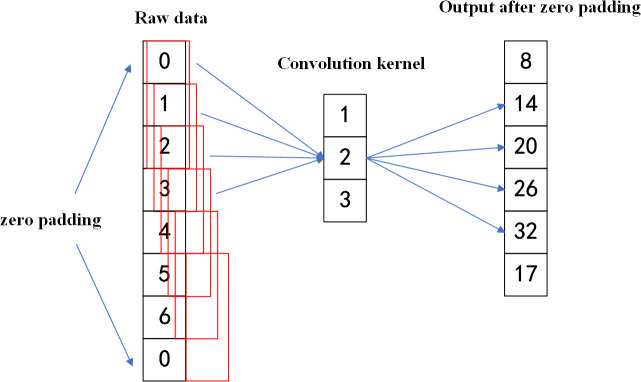
Zero Padding operation diagram. Zeros are added at both ends of one-dimensional data to maintain output length during convolution with kernel weights [1–3], preserving edge features.

During convolution, the kernel operates on local regions, which may result in the loss of edge information. Alrasheedi et al. [35] noted that padding is commonly used to mitigate this issue by extending the input at its boundaries. Among various techniques, zero padding is the most widely adopted. As Iftekharuddin et al. [36] pointed out, it preserves the input dimensions by adding zeros around the edges. A schematic of zero padding is shown in Fig 2.

(2) Batch Normalization (BN Layer)

Bilal et al. [37] described batch normalization (BN) as a technique that accelerates training and improves model stability, particularly in deeper networks where shifting activation distributions can lead to unstable gradients and slow convergence.

BN normalizes the inputs of each mini-batch by adjusting their mean and variance, followed by a learnable scaling and shifting operation to retain representational flexibility. This process mitigates vanishing or exploding gradients and enhances both convergence speed and generalization.

In convolutional neural networks (CNNs), the BN layer is typically applied after convolution. During training, it computes batch-wise statistics; during inference, it uses the mean and variance estimated during training for normalization.

Overall, BN is a powerful technique that significantly accelerates neural network training and enhances model performance by stabilizing gradient updates. The computation process of BN is described by Equations (2), (3), and (4), where the mean μ and variance $\sigma^{2}$ of all samples in a mini-batch are calculated as follows:


$$\begin{array}{c} \mu =\frac{1}{m}\sum_{i=1}^{m}\;x_{i},\sigma^{2}=\frac{1}{m}\sum_{i=1}^{m}\;{\left(x_{i}-\mu \right)}^{2} \end{array}$$ (2)

Normalized Data:


$$\begin{array}{c} {\hat{x}}_{i}=\frac{x_{i}-\mu }{\sqrt{\sigma^{2}+\in }} \end{array}$$ (3)

Linear Transformation:


$$\begin{array}{c} y_{i}=\gamma {\hat{x}}_{i}+\beta \end{array}$$ (4)

In the equations above, γ and β are learnable parameters used to restore the model’s representation capability.

The Batch Normalization (BN) layer significantly improves convergence speed, stabilizes training, and enhances generalization performance. It is commonly positioned after convolutional or fully connected layers, although its placement may vary depending on the specific network design.

(3) Activation Layer

Convolution operations are inherently linear, limiting their capacity to model complex patterns. To address this, activation functions introduce nonlinearity, enabling the network to learn more expressive feature representations through nonlinear transformations of the input.

An effective activation function should be nonlinear, differentiable, computationally efficient, and conducive to fast convergence. Its mathematical form is presented in Equation (5).


$$\begin{array}{c} y_{i}=f\left(x_{i+1}\right)=f\left(W_{i}⊗x_{i}+b_{i}\right) \end{array}$$ (5)

Common activation functions include Sigmoid, Tanh, ReLU, and their variants:

1) Sigmoid Function

The Sigmoid function is calculated as shown in Equation (6).


$$\begin{array}{c} f(x)=\frac{1}{1+e^{-x}} \end{array}$$ (6)

**Fig 3:**
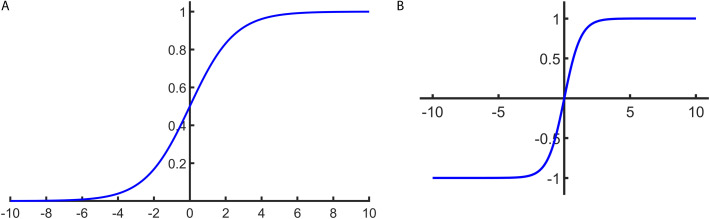
Sigmoid and Tanh activation function diagrams. (a) The sigmoid function, a bounded and differentiable curve that maps real-valued inputs into the range (0, 1). (b) The tanh function, which transforms inputs into the range (−1, 1), offering zero-centered output. Both functions exhibit sigmoid-like shapes and are widely used to introduce non-linearity in neural networks.

Elfwing et al. [38] reported that the Sigmoid activation function outputs values in the range [0, 1] but suffers from gradient saturation when inputs are extreme, causing vanishing gradients that hinder deep network training. They also noted its derivative is computationally expensive, limiting its use. The function’s schematic is shown in Fig 3(a).

2) Tanh Function

The Tanh function is calculated as shown in Equation (7).


$$\begin{array}{c} f(x)=\frac{e^{x}-e^{-x}}{e^{x}+e^{-x}} \end{array}$$ (7)

The Tanh function outputs values in the range [−1,1] and, unlike Sigmoid, is zero-centered, which facilitates optimization convergence. This centralized output benefits data processing. However, it also suffers from gradient saturation, causing vanishing gradients, and its derivative remains computationally complex. The function’s schematic is shown in Fig 3(b).

3) ReLU (Rectified Linear Unit) Function

The ReLU (Rectified Linear Unit) function is defined mathematically as shown in Equation (8).


$$\begin{array}{c} f(x)=max(0,x) \end{array}$$ (8)

The ReLU activation function is a piecewise linear function that maintains a linear output for positive inputs and exhibits a non-saturating property, effectively alleviating the vanishing gradient problem.

ReLU’s sparse activation characteristic enhances the model’s learning ability. However, if the network’s weights and learning rate are not set properly, ReLU may cause some neurons to get stuck in the negative half-region, preventing them from activation—a phenomenon known as the dying ReLU problem.

**Fig 4:**
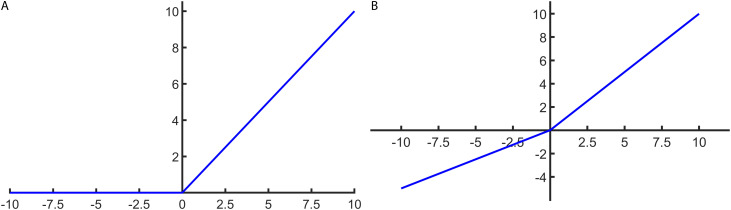
Relu and PRelu activation function diagram. (a) ReLU function, which outputs the input if positive, else zero. (b) PReLU function, a generalization of ReLU with a learned slope for negative inputs.

The function’s schematic diagram is shown in Fig 4(a).

4) PReLU (Parametric ReLU) Function

The Parametric ReLU (PReLU) function is mathematically defined as shown in Equation (9):


$$\begin{array}{c} y_{m,n}^{d}=\underset{i\in R_{m,n}^{d}}{max}x_{i} \end{array}$$ (9)

In the formula, γ is a learnable parameter.

Parametric ReLU (PReLU) extends ReLU by introducing a learnable parameter γ for the negative input region. For the ith neuron, negative inputs are scaled by this parameter instead of being set to zero.

This parametric form enables the network to adaptively learn the activation shape during training, improving expressiveness and generalization. PReLU is particularly effective for handling diverse data distributions by learning a more suitable activation function. The function’s schematic is shown in Fig 4(b).

(4) Pooling Layer

Pooling layers are commonly used in convolutional neural networks to reduce dimensionality and compress input data while retaining essential features. Typically applied after convolutional layers, pooling helps eliminate redundant information and highlights salient features, enhancing the network’s ability to learn.

**Fig 5:**
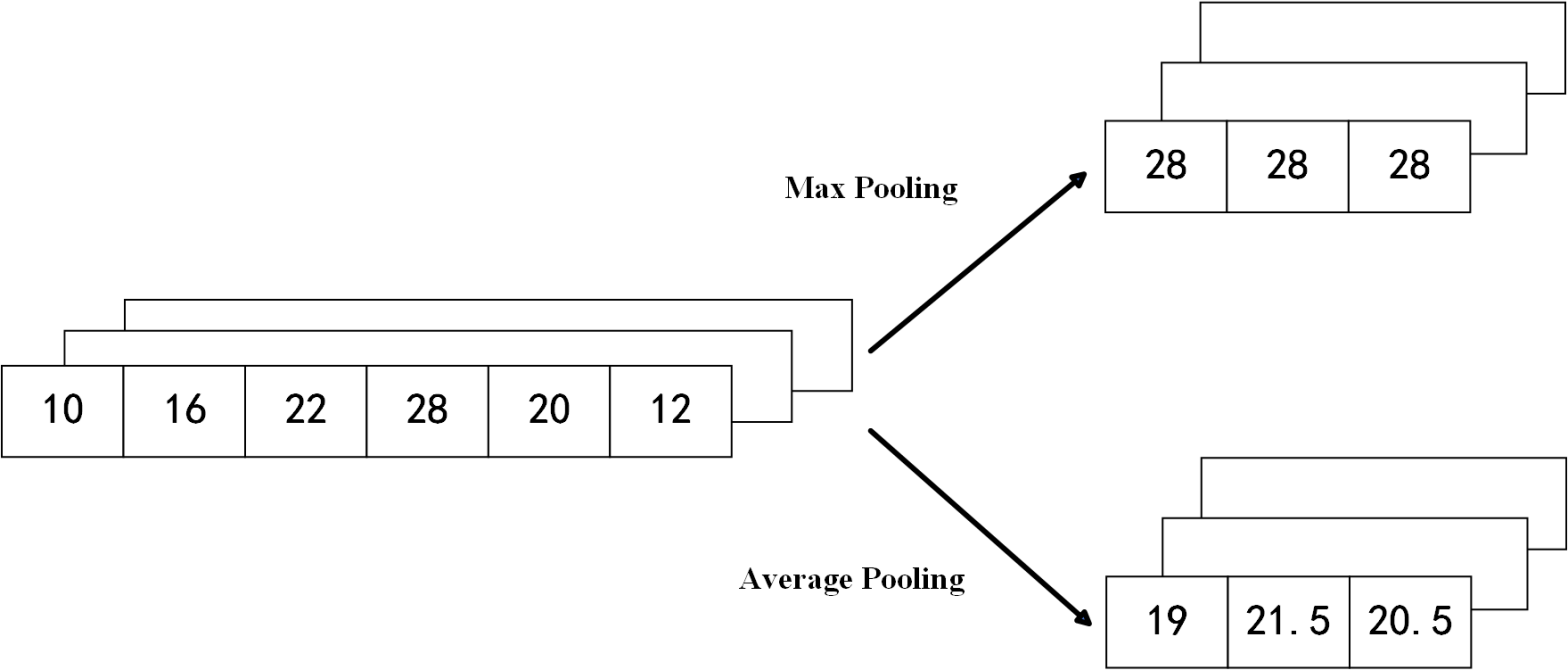
One-dimensional data pooling operation diagram. Max pooling selects the maximum value from each sliding window. Average pooling computes the mean value within each window. Both operations reduce data dimensionality while preserving key features.


Two main types of pooling are max pooling, which selects the maximum value within a region, and average pooling, which computes the average value. Their corresponding formulas are shown in Equations (10) and (11), with the operation illustrated in Fig 5.

$$\begin{array}{c} y_{m,n}^{d}=\underset{i\in R_{m,n}^{d}}{max}x_{i} \end{array}$$ (10)

$$\begin{array}{c} y_{m,n}^{d}=\frac{1}{\left|R_{m,n}^{d}\right|}\sum_{i\in_{m,n}^{d}}x_{i} \end{array}$$ (11)

The pooling layer slides a fixed-size window over the input with a set stride. Within each window, it aggregates data into a single value, typically using max pooling or average pooling depending on the method applied.

(5) Fully Connected Layer

**Fig 6:**
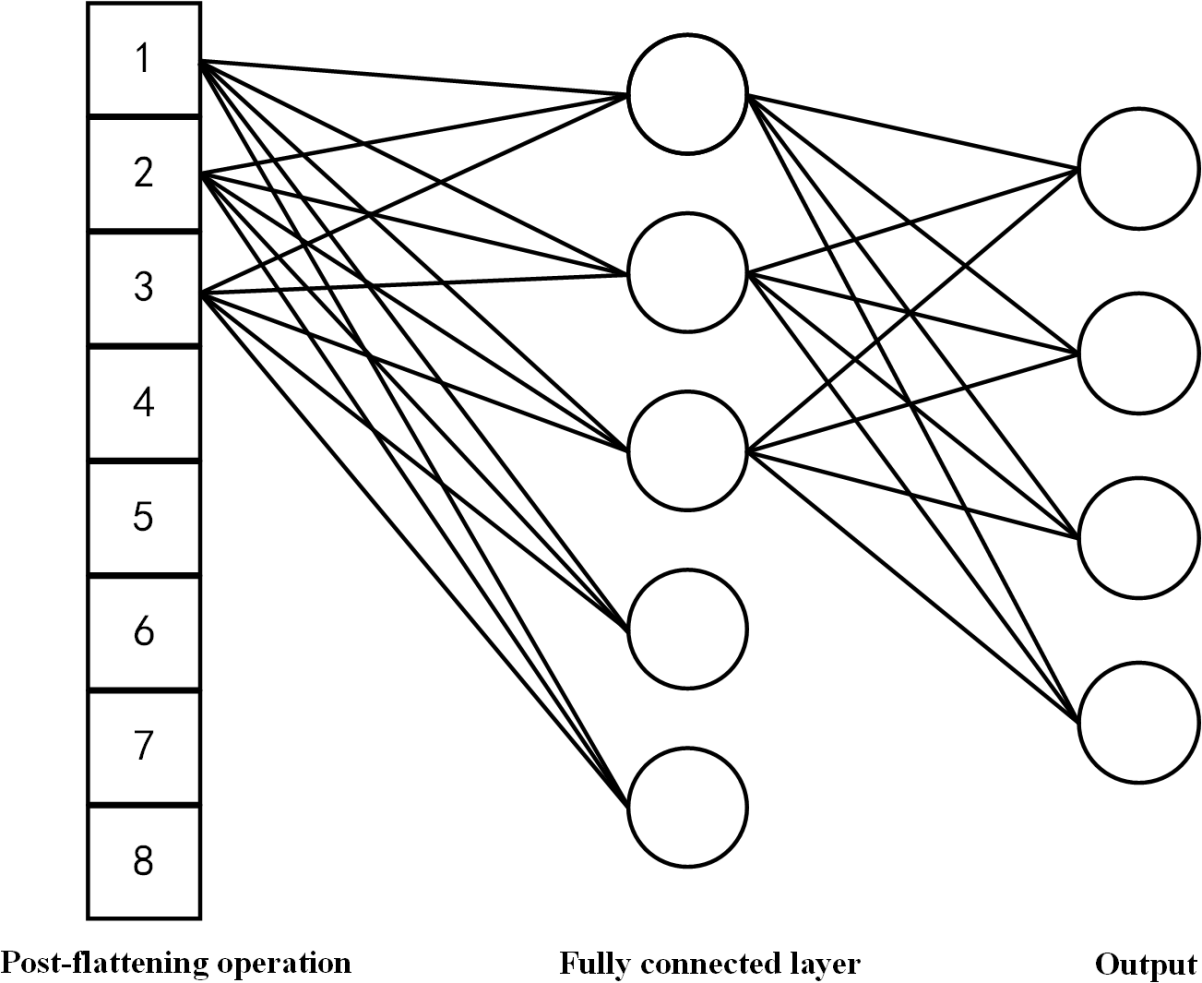
Diagram of fully connected layer operation for one-dimensional data. Illustrates how flattened one-dimensional data is processed through a fully connected neural network layer. Each node in the fully connected layer receives input from all nodes in the previous layer, transforming the data through learned weights to produce the output.


The fully connected (FC) layer, positioned at the final stage of a convolutional neural network, transforms extracted features into the class space of input samples. The output is computed via the Softmax function, as shown in Equations (12) and (13). A schematic of the FC layer operation is presented in Fig 6.

$$\begin{array}{c} y_{i}=\sum_{j=1}^{n}\;w_{ij}x_{j}+b_{i} \end{array}$$ (12)

$$\begin{array}{c} p\left(y=j|x\right)=\frac{e^{y_{j}}}{\sum_{k=1}^{K}\;e^{y_{k}}} \end{array}$$ (13)

In these equations:

$y_{i}$ represents the output of the fully connected layer.

p(y=j|x) denotes the Softmax output, which represents the probability of the input x belonging to class j.

Krepresents the total number of classes.

Features from the final pooling layer are first flattened into a one-dimensional vector before being input to the fully connected (FC) layer. In this layer, the output is produced by a weighted sum of all inputs, followed by an activation function. The FC layer integrates local features extracted by convolutional and pooling layers, forming a fully connected network structure.

### 2.2 Propagation process of convolutional neural networks

Morsy et al. [39] and Yamashita et al. [40] stated that convolutional neural networks (CNNs) rely on forward propagation and backpropagation to optimize network parameters, enabling effective feature extraction and classification. These processes are crucial for refining parameters and improving the model’s ability to distinguish fault patterns in rolling bearing diagnosis. Forward propagation passes input data through the network layers to compute feature representations and predictions, while backpropagation updates parameters based on error feedback to enhance training effectiveness.

In rolling bearing vibration analysis, signals contain rich fault-related information. CNNs utilize multi-layer convolutions to extract meaningful features and suppress redundancy, thereby improving diagnostic accuracy.

The key steps of forward propagation include:

1. Convolutional Layer: Extracts local features via convolution, described by Equation (14).

$$\begin{array}{c} y[i]=\sum_{m=1}^{M}\;\sum_{n=1}^{N}\;x[m,n]\cdot w[i-m+1,i-n+1] \end{array}$$ (14)

In these equation,x[m,n] represents the input data, and w[i−m+1,i−n+1] denotes the convolution kernel.

2. Activation Function: Performs nonlinear mapping to enhance feature representation capability. Common activation functions are expressed as shown in Equation (15).

$$\begin{array}{c} f(x)=max(0,x) \end{array}$$ (15)

3. Pooling Layer: Reduces data dimensions, decreases computational complexity, and retains key features. A commonly used pooling method is max pooling, with its computation formula shown in Equation (16).

$$\begin{array}{c} y[i]=max\left(x[i-k+1:i+k]\right) \end{array}$$ (16)

4. Fully Connected Layer: Maps the extracted features to fault categories and computes the final output using Softmax, with its computation formula shown in Equation (17).

$$\begin{array}{c} p\left(y=j|x\right)=\frac{e^{z_{j}}}{\sum_{k=1}^{K}\;e^{z_{k}}}\; \end{array}$$ (17)

In these equation, $z_{j}$ represents the output of the fully connected layer, and K denotes the number of categories.

Backpropagation is a method used to compute the gradient of the loss function with respect to each weight and bias, enabling the update of network parameters. The core steps are as follows.

1. Error Calculation: The difference between the predicted value and the actual value is measured by the loss function, with the specific computation formula shown in Equation (18).

$$\begin{array}{c} L\left(y,\hat{y}\right)=-\sum_{i=1}^{N}\;y_{i}\text{log}\left({\hat{y}}_{i}\right) \end{array}$$ (18)

2. Error Backpropagation: The error is propagated backward through the network from the output layer to the input layer, gradually computing the gradient of each parameter. The specific computation formula is shown in Equation (19).

$$\begin{array}{c} \frac{\partial L}{\partial w_{ij}}=\frac{\partial L}{\partial z_{j}}\cdot \frac{\partial z_{j}}{\partial w_{ij}} \end{array}$$ (19)

3. Parameter Update: The stochastic gradient descent (SGD) algorithm is used to adjust the weights based on the computed gradients to minimize the loss. The specific computation formula is shown in Equation (20).

$$\begin{array}{c} w_{ij}=w_{ij}-\eta \cdot \frac{\partial L}{\partial w_{ij}} \end{array}$$ (20)

In the equation, η represents the learning rate.

**Fig 7:**
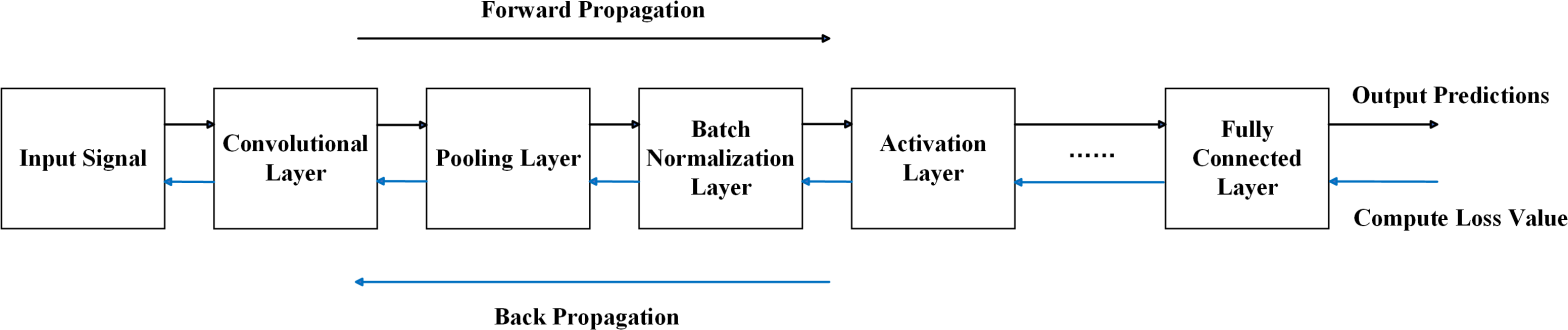
Convolutional neural network forward and backpropagation processes. The figure illustrates the forward propagation from input through convolutional, pooling, normalization, activation, and fully connected layers to produce predictions, followed by backpropagation that computes loss and updates weights across the network.

Forward propagation and backpropagation are fundamental to CNN training. Forward propagation computes outputs, allowing the model to predict data categories, while backpropagation adjusts parameters to enhance accuracy. Through iterative interaction, the CNN progressively improves its fault diagnosis performance. These processes are illustrated in Fig 7.

### 2.3 One-dimensional convolutional neural network model

Convolutional neural networks (CNNs) are primarily designed for image processing using two-dimensional (2D) convolutional architectures to extract spatial features. To apply 2D CNNs to one-dimensional (1D) vibration signals, researchers often reshape 1D data into 2D matrices or transform signals into time-frequency representations via wavelet or fast Fourier transforms (FFT).

**Fig 8:**
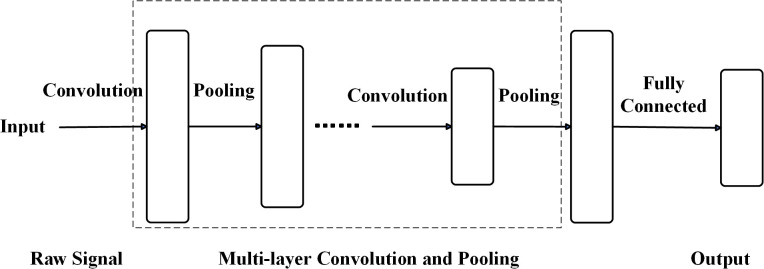
Traditional one-dimensional convolutional neural network model. The model processes raw one-dimensional signals through sequential convolution and pooling layers for feature extraction, followed by a fully connected layer for final output prediction.

While these approaches improve fault diagnosis, they incur high computational cost and complexity. In contrast, 1D CNNs offer lower computational overhead and training time. The traditional 1D CNN architecture is illustrated in Fig 8.

One-dimensional convolutional neural networks (1D-CNNs) are designed for time series data and can be viewed as simplified versions of two-dimensional CNNs (2D-CNNs). While 2D-CNN kernels typically have dimensions of k×k, 1D-CNN kernels reduce the height dimension, resulting in k×1 kernels.

The operation of 1D-CNNs parallels that of 2D-CNNs. Since rolling bearing vibration signals are one-dimensional time series, 1D-CNNs are better suited for fault diagnosis in this context.

### 2.4 Parallel one-dimensional convolutional neural network model

Although one-dimensional convolutional neural networks (1D-CNNs) effectively process time series signals, they rely solely on time-domain information, limiting feature extraction. Li et al. [41] proposed a parallel 1D-CNN (P1D-CNN) model to incorporate frequency-domain information more comprehensively.

This model employs two channels with convolution kernels of different sizes in the first layer. Subsequent layers use smaller kernels, enabling deeper architectures, enhancing local feature capture, and reducing training time. Sun et al. [42] noted that larger kernels have wider receptive fields, making them better suited for extracting global features.

**Fig 9:**
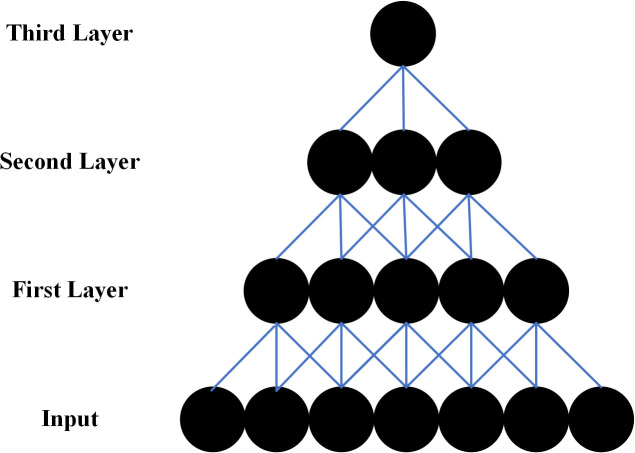
Schematic diagram of receptive field. Depicts how the receptive field expands hierarchically across layers, with each neuron processing increasingly broader regions of the input through connections to previous layers.

The receptive field denotes the input region influencing each output neuron in a CNN layer. For a 1D convolutional layer with kernels of width k, each neuron processes a local segment of length k. Fig 9 illustrates this concept.

**Fig 10:**
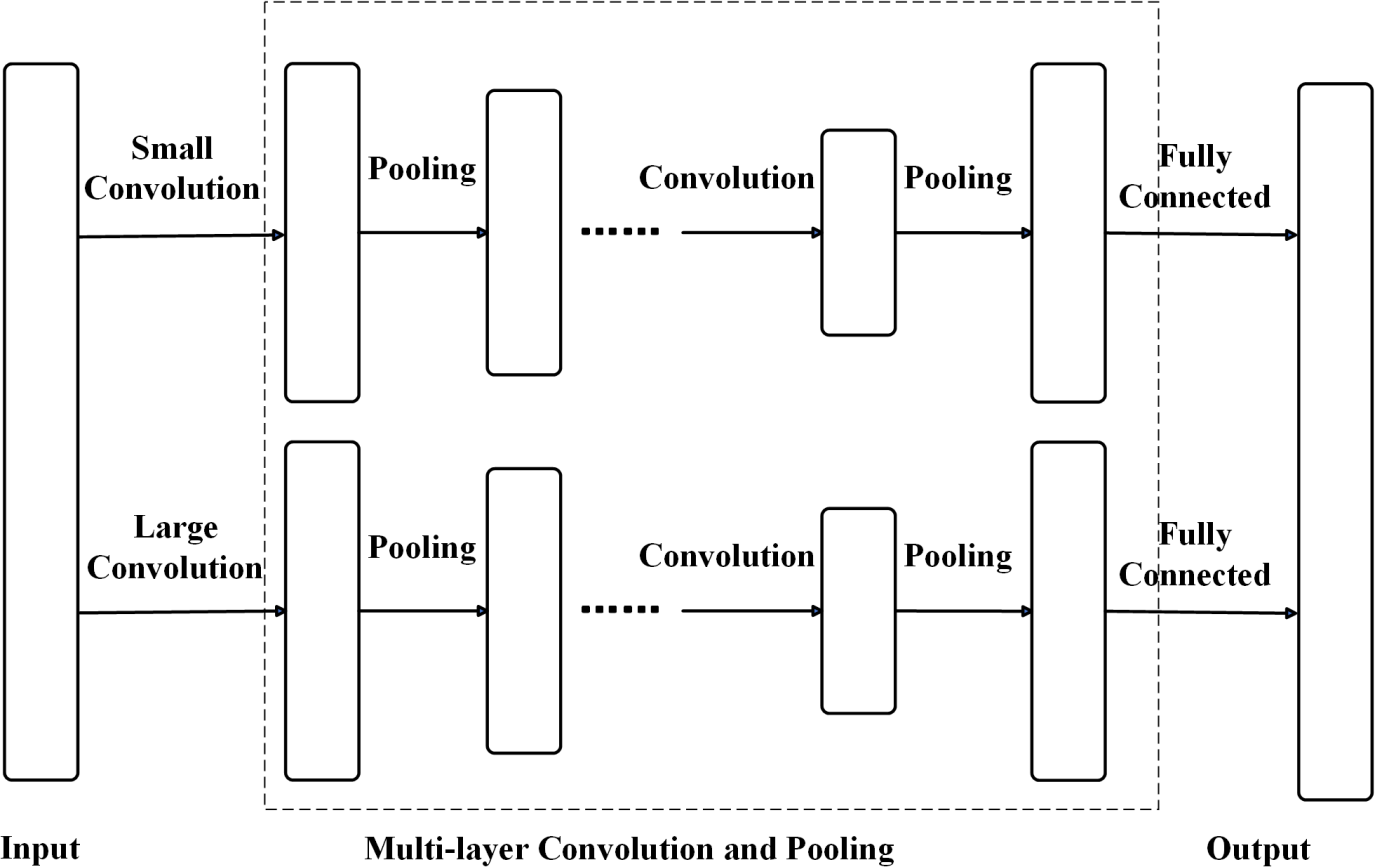
Parallel one-dimensional convolutional neural network model. Shows two separate convolution and pooling pathways with distinct kernel sizes processing the same input. The outputs merge at the fully connected layer, enabling multi-scale feature extraction.

Compared to single-channel 1D-CNNs, the parallel 1D-CNN (P1D-CNN) effectively extracts both time- and frequency-domain features from vibration signals while accelerating network training. The architecture of the P1D-CNN is shown in Fig 10.

### 2.5 Improved parallel one-dimensional convolutional neural network model

#### 2.5.1 Gated recurrent unit.

Hochreiter et al. [43] proposed the Long Short-Term Memory (LSTM) network in 1997.However, due to the limitations of computing power and dataset size at the time, it did not gain widespread application. In 2014, Cho et al. [44]introduced the Gated Recurrent Unit (GRU), an improved recurrent neural network (RNN) architecture.

GRU effectively addresses the vanishing gradient problem in traditional RNNs and reduces computational complexity by incorporating two gating mechanisms: the reset gate $r_{t}$ and the update gate $z_{t}$ These gate vectors determine which information should be retained or discarded, allowing the model to capture long-term dependencies in time series data more effectively.

At time step t the reset gate $r_{t}$ and the update gate $z_{t}$ are computed using the following equations, as shown in Equations (21) and (22):


$$\begin{array}{c} z_{t}=\sigma \left(W_{z}\cdot \left[h_{t-1},x_{t}\right]+b_{z}\right) \end{array}$$ (21)

$$\begin{array}{c} r_{t}=\sigma \left(W_{r}\cdot \left[h_{t-1},x_{t}\right]+b_{r}\right) \end{array}$$ (22)

In these equations, σ represents the Sigmoid activation function, while $W_{z}$ and$W_{r}$ are learnable weight matrices. $h_{t-1}$ denotes the hidden state from the previous time step, and represents the current input features.

The reset gate $r_{t}$ determines whether the network should ignore previous state information, while the update gate $z_{t}$ controls the degree to which new information is accepted. By regulating the interaction between the current input and the previous hidden state, the reset gate $r_{t}$ helps compute the candidate hidden state ${\tilde{h}}_{t}$, which is formulated as shown in Equation (23):


$$\begin{array}{c} {\tilde{h}}_{t}=\tan\text{h}\left(W\cdot \left[r_{t}*h_{t-1},x_{t}\right]+b\right) \end{array}$$ (23)

The final hidden state $h_{t}$ is computed using the update gate $z_{t}$ and the candidate state ${\tilde{h}}_{t}$, as shown in Equation (24):


$$\begin{array}{c} h_{t}=\left(1-z_{t}\right)*h_{t-1}+z_{t}*{\tilde{h}}_{t} \end{array}$$ (24)

GRU addresses the vanishing gradient issue in traditional RNNs by using reset and update gates to regulate information flow. The reset gate $r_{t}$ controls the extent to which previous state information is ignored, while the update gate $z_{t}$ determines how much new information is retained. This gating structure allows the network to dynamically manage information, preserving key features and improving time series representation.

In rolling bearing fault diagnosis, where vibration signals exhibit complex temporal patterns and long-term dependencies, GRU effectively captures these characteristics, enhancing diagnostic performance.

#### 2.5.2 Attention mechanism.

Bahdanau et al. [45] introduced the attention mechanism, which enables models to focus selectively on important parts of the input, rather than treating all inputs equally. This mechanism effectively mitigates performance degradation caused by long-range dependencies in traditional recurrent neural networks. Unlike fixed-weight or fully connected methods, the attention mechanism dynamically learns weights, reducing parameter count and improving both efficiency and generalization.

**Fig 11:**
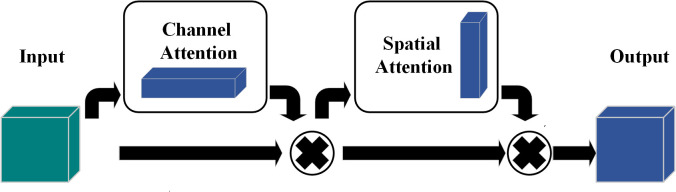
CBAM attention mechanism. Depicts the sequence of modules: input, channel attention, spatial attention, and output. CBAM efficiently enhances network performance while reducing parameters and computation, and can be integrated into various networks.

Woo et al. [46] proposed the Convolutional Block Attention Module (CBAM), a lightweight attention module that applies attention sequentially along spatial and channel dimensions. This study adopts CBAM due to its effectiveness in enhancing feature representation. The CBAM module operates in two steps, with formulas given in Equations (25) and (26), and its schematic is shown in Fig 11.

1. Channel Attention (ChannelAttention): The input feature F undergoes max pooling and average pooling operations, producing the max-pooled feature $F_{\max}$ and average-pooled feature $F_{avg}$ These features are concatenated and passed through a shared fully connected layer, generating the channel attention weight $A_{c}$, with its computation process given in Equation (25).

$$\begin{array}{c} \;A_{c}=\sigma \left(FC\left(ReLU\left(FC\left(\left[F_{max},F_{avg}\right]\right)\right)\right)\right)) \end{array}$$ (25)

In the equation, σ represents the Sigmoid activation function, and FC denotes the fully connected layer operation.

Then, the channel attention weight FC is element-wise multiplied with the input feature F to obtain the adjusted feature $F^{\prime }$. Its computation is given by Equation (26).


$$\begin{array}{c} F^{\prime }=M_{c}(F)⊗F \end{array}$$ (26)

2. Spatial Attention (Spatial Attention): The channel attention-adjusted feature $F^{\prime }$ undergoes max pooling and average pooling along the channel dimension, producing the spatial features $F_{\max}^{\prime }$ and $F_{avg}^{\prime }$.These two features are concatenated and processed through a convolutional layer to generate the spatial attention weight $A_{s}$. Its computation process is given by Equation (27).

$$\begin{array}{c} A_{s}=\sigma \left(\text{Conv}\left(\text{ReLU}\left(\text{Conv}\left(\left[F_{max}^{\prime },F_{\text{avg}}^{\prime }\right]\right)\right)\right)\right) \end{array}$$ (27)

The Sigmoid activation function is used to constrain the spatial attention weight $A_{s}$ within the range of 0–1.

Finally, the spatial attention weight $A_{s}$ is applied to the adjusted feature $F^{\prime }$ to obtain the final feature representation $F^{\prime \prime }$. Its computation is given by Equation (28).


$$\begin{array}{c} F^{\prime \prime }=M_{s}\left(F^{\prime }\right)⊗F^{\prime } \end{array}$$ (28)

The attention mechanism dynamically learns feature weights, automatically selecting the most relevant features from the input data and thereby enhancing the model’s feature selection capability. In rolling bearing fault diagnosis, vibration signals often contain substantial noise and redundant information. The attention mechanism effectively filters out these irrelevant components, significantly improving diagnostic accuracy. Moreover, by reducing redundant parameters, it enhances the model’s efficiency and generalization ability. This is particularly beneficial when processing complex vibration signals, as the attention mechanism helps the model adapt to varying data distributions and strengthens its overall generalization performance.

As shown in Fig 11, CBAM (Convolutional Block Attention Module) is a structure composed of input, channel attention module, spatial attention module, and output. This model not only saves parameters and reduces computational complexity but can also be flexibly embedded into existing network architectures for optimization. By leveraging channel attention and spatial attention mechanisms, CBAM can significantly enhance the network’s performance while maintaining computational efficiency.

#### 2.5.3 Fault diagnosis process based on improved parallel one-dimensional convolutional neural network.

**Fig 12:**
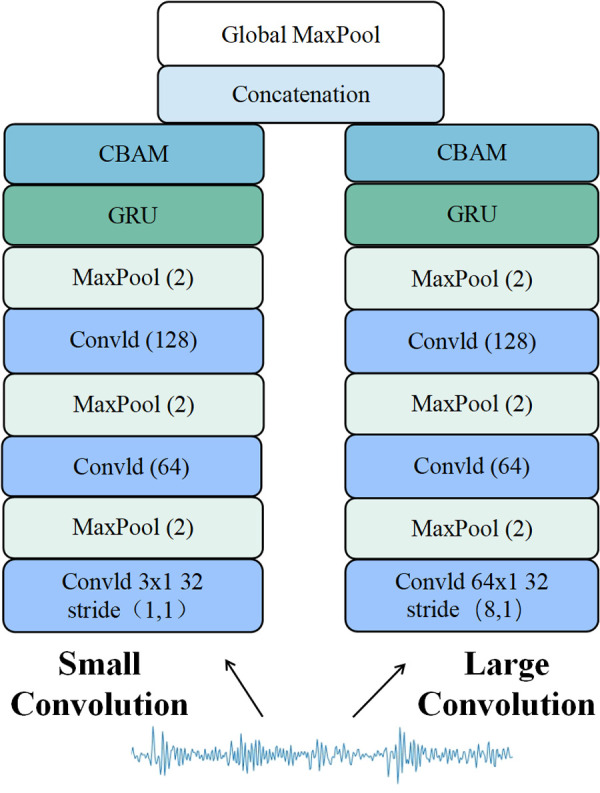
Improved one-dimensional convolutional neural network model. Features dual convolutional pathways with different kernel sizes for multi-scale feature extraction. Each pathway includes CBAM attention and GRU layers to enhance feature and temporal learning. Outputs are combined after global max pooling to generate refined representations.

The parallel one-dimensional convolutional neural network (1D-CNN) is a deep convolutional neural network. As the network depth increases, it may suffer from the vanishing gradient problem. To address this issue, GRU (Gated Recurrent Unit) and an attention mechanism are introduced into the classifier module. GRU effectively mitigates the vanishing gradient and long-term dependency issues in recurrent neural networks by incorporating reset and update gates. However, when processing signals, GRU tends to forget information once the signal exceeds a certain length. To resolve this, an attention mechanism is introduced to compress the data, alleviating GRU’s forgetting problem and enabling the model to quickly pinpoint key information within a large dataset. The improved parallel one-dimensional convolutional neural network model structure is shown in Fig 12.

**Fig 13:**
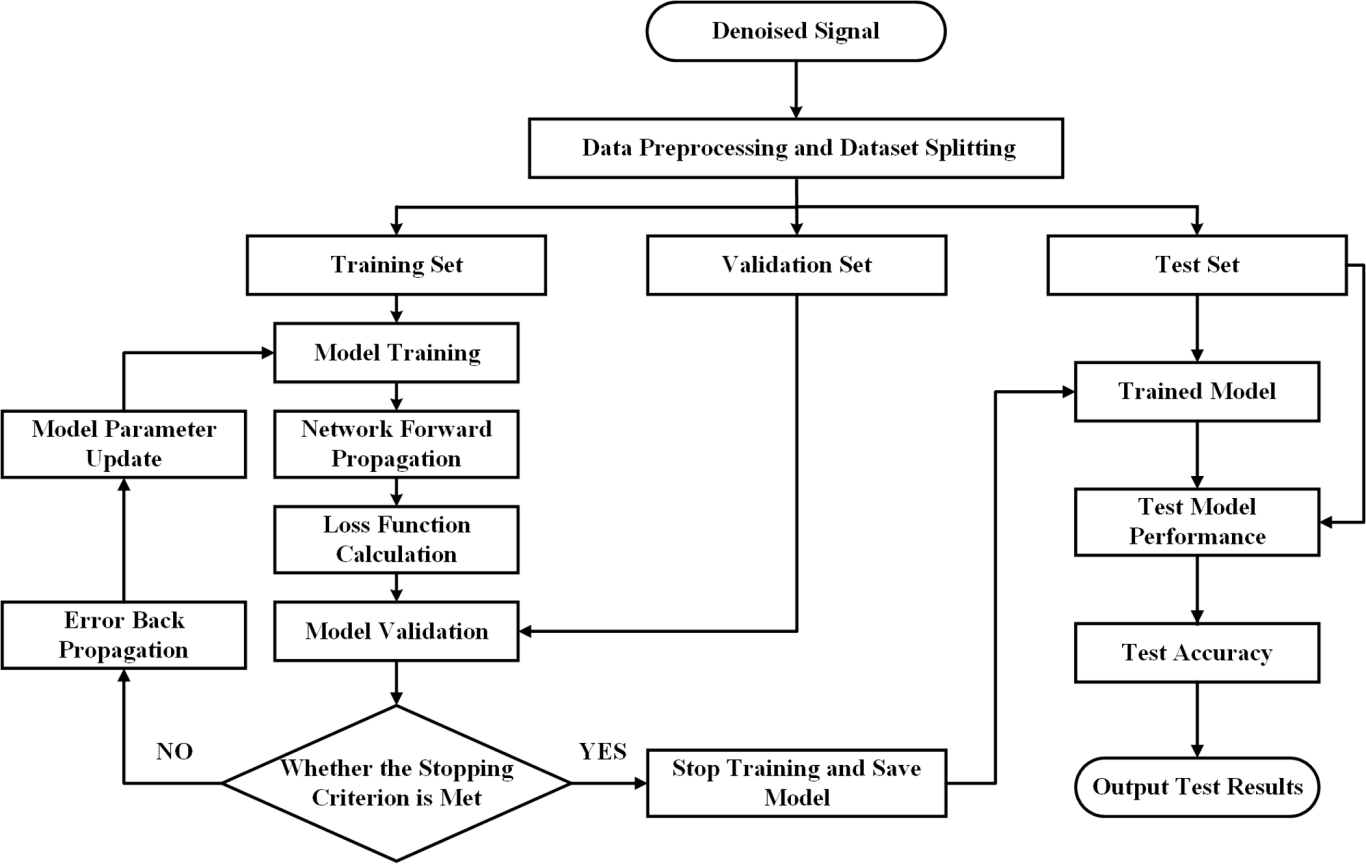
Improved one-dimensional convolutional neural network fault diagnosis flowchart. The process includes data preprocessing, dataset splitting, model training and validation, and final testing to evaluate diagnostic accuracy.

As shown in Fig 12, the improved parallel one-dimensional convolutional neural network (1D-CNN) model adopts a dual-channel convolutional kernel of different sizes to process vibration signals. The two channels separately extract the time-domain and frequency-domain features of the vibration signal, which not only accelerates the training speed of the network model but also fully utilizes the signal’s characteristic information. After extracting the vibration signal features through three convolutional and pooling layers, the GRU layer performs temporal modeling on the extracted features, learning the relationships and dynamic changes within the sequence. The model generates a large number of high-dimensional temporal features, which are often difficult to distinguish and interpret intuitively. By introducing an attention mechanism layer after the GRU layer, the model can extract important information, allowing it to quickly identify key points from a vast amount of data while filtering out irrelevant information, making the training process more efficient. Finally, a global max pooling layer replaces the fully connected layer, which reduces the number of parameters, improves computational efficiency, and lowers the risk of overfitting. The fault diagnosis process of the improved parallel 1D-CNN is illustrated in Fig 13.

As shown in Fig 13, the fault diagnosis algorithm process is as follows:

1. Preprocessing the Vibration Signal:
  The original vibration signal is decomposed, and the relevant components are selected and reconstructed.
  The reconstructed signal is then divided into training, testing, and validation sets.
2. Building the Improved Parallel 1D-CNN Fault Diagnosis Model:
  A dual-channel multi-layer convolutional and pooling network is constructed.
  A Gated Recurrent Unit (GRU) is introduced to optimize the network model, reducing the risk of gradient explosion in deep networks.
  An attention mechanism is incorporated to address the forgetting issue that may arise in GRU during backpropagation.
  A global max pooling layer is used to prevent overfitting during training.
  Finally, a Softmax layer is applied for classification output, completing the fault diagnosis model setup.
3. Model Training and Validation:
  The training dataset is fed into the improved 1D-CNN model for forward propagation and loss function computation.
  The validation set is then input into the model for performance evaluation.
  The output results are compared with the ground truth, and the error is calculated.
  If the stopping criterion is met, the training terminates, and the model is saved.
  If the criterion is not met, error backpropagation updates the parameters of each network layer, and training continues until the termination condition is satisfied.
4. Model Testing and Evaluation:
  The testing dataset is fed into the trained model.
  The model outputs a confusion matrix for the test set to validate the diagnostic results.

## 3. Experimental verification and analysis

### 3.1 Experimental verification and result analysis based on public datasets

To verify the effectiveness of the improved one-dimensional convolutional neural network (1D-CNN) algorithm proposed in this paper, the Case Western Reserve University (CWRU) bearing dataset was selected for experimental analysis. This dataset is a widely used benchmark in the field of rolling bearing fault diagnosis and can effectively evaluate the reliability and accuracy of mechanical fault diagnosis algorithms.

**Fig 14:**
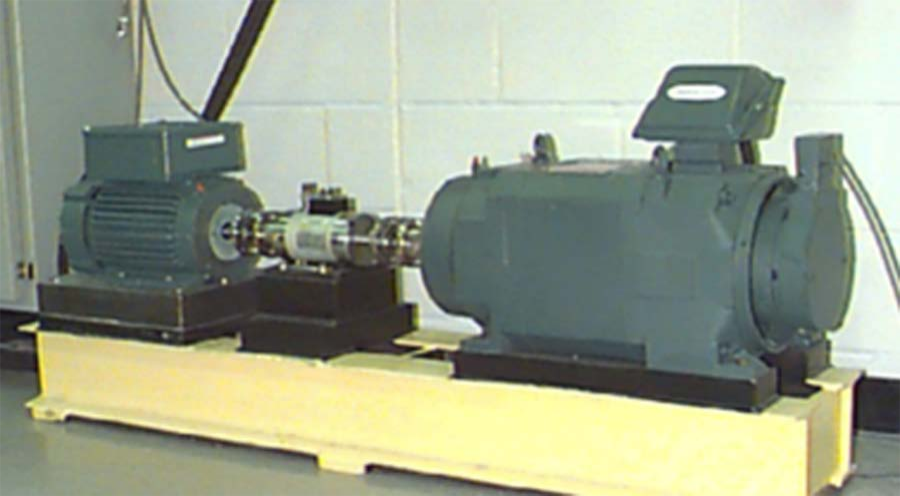
Experimental platform for CWRU bearing vibration dataset. Photograph of the experimental setup for collecting bearing vibration data. [Source: Case Western Reserve University Bearing Data Center].

In the experiment, a two-horsepower motor was used for fault testing. Single-point faults of different diameters were introduced at various locations of the motor, and vibration signals were collected using sensors mounted on the motor housing. The detailed configuration of the fault testing platform is shown in Fig 14.

The CWRU dataset covers various fault types and operating conditions, including inner ring faults, outer ring faults, rolling element faults, and normal states. To comprehensively evaluate the performance of the model, the signal sampling frequency was set to 12 kHz, and the rotational speed was 1797 r/min. Data from 10 categories were selected, including one class of normal vibration signals and nine classes of fault vibration signals. The fault defect diameters were 0.1778 mm, 0.3556 mm, and 0.5334 mm. The dataset sample length was set to 1200, and the dataset was divided into training, testing, and validation sets in a ratio of 7:2:1. Table 1 presents the detailed classification of bearing data labels, including the bearing condition, fault diameter, number of samples, and corresponding class labels.

**Table 1:** Classification of bearing data labels.

Bearing Condition	Single Point Diameter/mm	Number of Samples	Class Label
**Inner Race**	0.1778	605	0
**Outer Race**	0.1778	600	1
**Roller**	0.1778	600	2
**Inner Race**	0.3556	605	3
**Outer Race**	0.3556	605	4
**Roller**	0.3556	605	5
**Inner Race**	0.5334	605	6
**Outer Race**	0.5334	605	7
**Roller**	0.5334	605	8
**Normal**	0.1778	2420	9

The dataset includes ten bearing conditions collected at 1797 r/min with a sampling frequency of 12 kHz. Fault classes correspond to inner race, outer race, and roller defects with diameters of 0.1778 mm, 0.3556 mm, and 0.5334 mm. Each sample contains 1200 data points.

Using TensorFlow-GPU (version 2.0.0) and Python (version 3.6.3), we executed and implemented the model. The computer processor (CPU) used was an Intel(R) Core(TM) i7-13620H, and the graphics card (GPU) was an NVIDIA GeForce RTX 4060.To better demonstrate the advantages of the model, we optimized the performance of the network model. The parameter metrics are shown in Table 2.

**Table 2:** Model parameter indicators.

Parameter	Parameter Setting
**Learning Rate**	le-5
**Dropout**	0.5
**Activation Function**	Relu
**Batch Size**	16
**Iterations**	100

The model parameters include a learning rate of 1e-5, a dropout rate of 0.5, and the ReLU activation function. Training was conducted with a batch size of 16 over 100 iterations.

The learning rate controls the size of parameter updates in each step, ensuring a more stable and reliable training process. Dropout randomly deactivates certain neuron activations during training, helping to prevent overfitting. The ReLU activation function is used to measure model performance and guide optimization, further mitigating overfitting. Batch size determines the number of samples used to update model parameters during iterative training, enhancing the model’s generalization ability and stability. Properly setting the number of iterations effectively trains a high-performance model with strong generalization capabilities.

**Fig 15:**
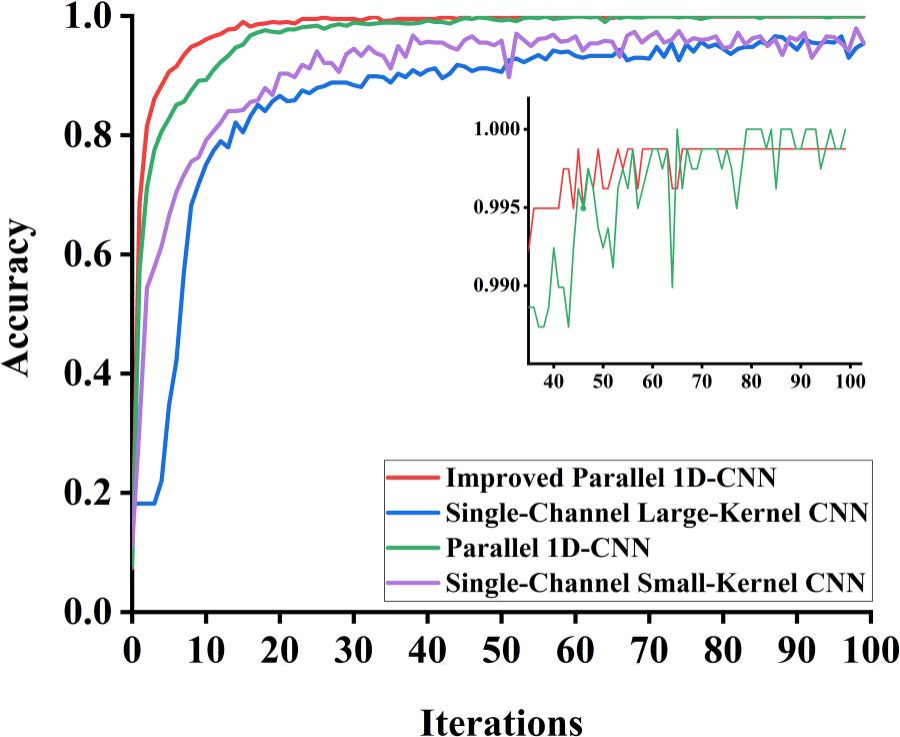
Accuracy chart of each model validation set. The improved parallel 1D-CNN model achieves higher accuracy and greater stability than other models. The inset zooms in on accuracy fluctuations in the later training iterations.

The preprocessed CWRU public dataset underwent noise reduction before being divided into training, testing, and validation sets in a 7:2:1 ratio. The training set samples were fed into the improved parallel 1D-CNN model for training, where forward propagation was performed to compute the loss function. The validation set samples were then input into the model for validation. For comparison, single-channel large-kernel CNN, single-channel small-kernel CNN, and parallel 1D-CNN models were selected alongside the proposed algorithm in this chapter. The accuracy variation curves of the validation sets for each model during training are shown in Fig 15.

**Fig 16:**
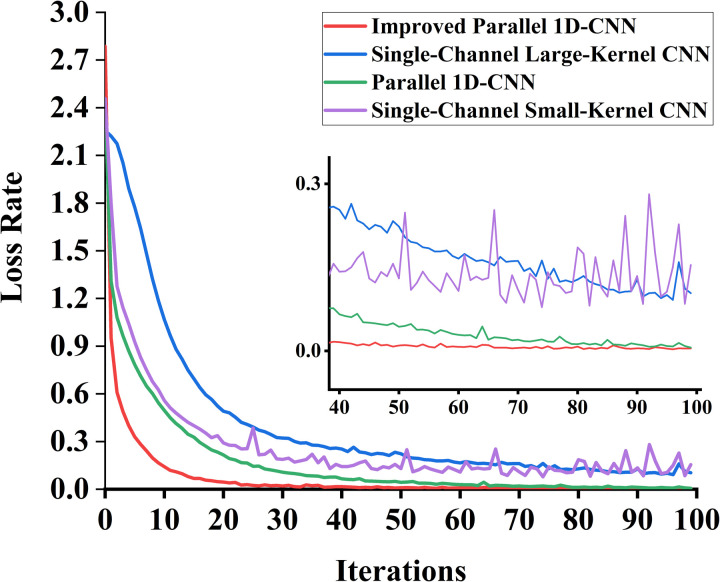
Loss rate graph of each model validation set. The improved parallel one-dimensional CNN model achieves a faster decrease in loss and maintains higher stability compared to other models. The inset highlights detailed loss variations during the final training stages.

From Fig 15, it can be observed that the accuracy variation curve of the dual-channel CNN network is more stable and achieves higher accuracy compared to the single-channel CNN network. Compared to the parallel 1D-CNN, the proposed algorithm in this chapter converges faster, reaching a stable accuracy after 20 training epochs, which verifies the model’s accuracy and stability. The loss variation curves of the validation sets for each model during training are shown in Fig 16.

**Fig 17:**
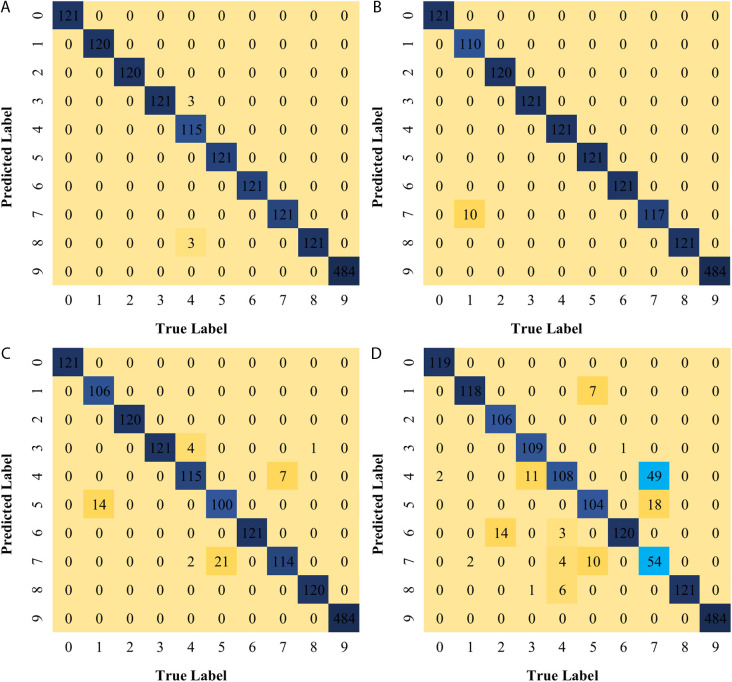
Accuracy of test sets for each model, specifically. The matrices show classification performance for four models: (a) Improved Parallel 1D-CNN, (b) Single-Channel Large-Kernel CNN, (c) Parallel 1D-CNN, and (d) Single-Channel Small-Kernel CNN. Each matrix presents the counts of correct and incorrect predictions across classes, enabling direct comparison of model accuracy.

The segmented test set samples were input into the trained improved parallel 1D-CNN fault diagnosis model for validation, and the resulting test set accuracy is shown in Fig 17(a). From the figure, the horizontal axis represents the number of actual labels, while the vertical axis represents the number of predicted labels. Based on the label correspondence, it can be observed that the proposed algorithm in this chapter achieves high accuracy on the test set. The same dataset samples were then tested using the parallel 1D-CNN, single-channel small-kernel CNN, and single-channel large-kernel CNN models. The corresponding results are shown in Figs 17(b), 17(c), and 17(d), respectively. By comparison, it is evident that the proposed model in this chapter achieves significantly higher accuracy in diagnosing the test set than the other models.

By organizing the confusion matrices mentioned above, the accuracy of different fault diagnosis models was obtained, as shown in Table 3. It can be seen that the proposed algorithm in this paper achieves an accuracy of 99.62%, which is higher than that of other models. Compared to the small-kernel CNN, the accuracy of the large-kernel CNN decreases. This is mainly because small kernels have fewer parameters, enabling better parameter sharing, making the model more compact, and reducing the risk of overfitting. The dual-channel network achieves higher accuracy than the single-channel network, confirming that the dual-channel structure can more comprehensively capture feature information, thereby enhancing the model’s recognition accuracy. The proposed algorithm in this chapter shows a significant improvement in accuracy compared to the dual-channel convolutional neural network model. This indicates that the proposed algorithm can fully utilize feature information, reduce training time while improving model accuracy, and verify the effectiveness of the model.

**Table 3:** Accuracy of test set for different fault diagnosis models.

Fault Diagnosis Model	Accuracy
**Single-Channel Large-Kernel CNN**	91.85%
**Single-Channel Small-Kernel CNN**	96.88%
**Parallel 1D-CNN**	99.11%
**Improved Parallel 1D-CNN**	99.62%

Test set accuracy of different fault diagnosis models used for bearing condition classification. The improved parallel 1D-CNN demonstrates the highest accuracy among the compared models.

To further verify the generalization ability of the proposed method, in addition to using the publicly available CWRU dataset, experiments were also conducted on the publicly available Southeast University dataset. This dataset includes a wider range of fault types and operating conditions, allowing for a more comprehensive evaluation of the model’s performance. The experimental results show that the proposed model achieves an average diagnostic accuracy of 99.62% on the CWRU dataset and an average diagnostic accuracy of 98.7% ± 0.3% (95% confidence interval) on the other public dataset. This represents a 5.2 percentage point improvement over the traditional CNN-GRU hybrid model, further demonstrating its effectiveness and generalization ability.

### 3.2 Experimental verification and result analysis based on measured vibration signals from a test bench

#### 3.2.1 Performance comparison of representative conventional methods.

**Fig 18:**
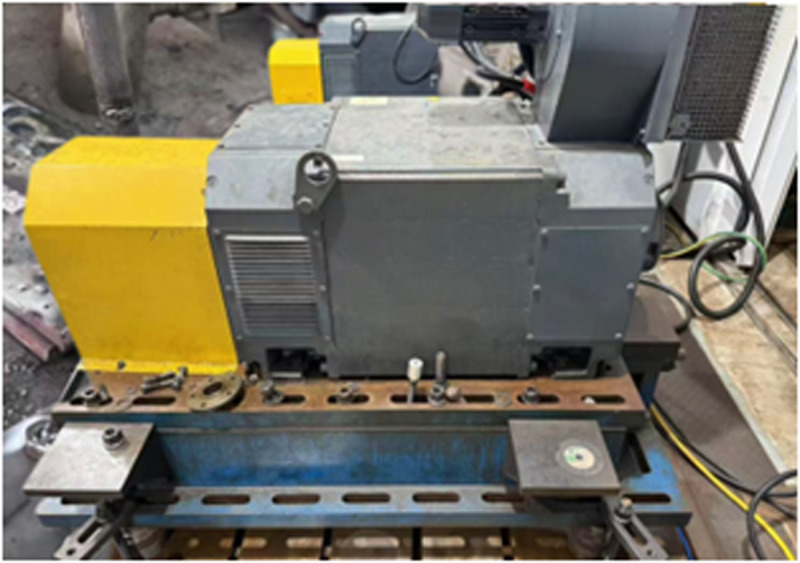
The self-built bearing vibration data acquisition experimental platform. The platform comprises a motor, bearing housing, and data acquisition system, designed to capture vibration signals from bearings under various operating conditions.

To verify the effectiveness of the proposed improved method, experimental analysis was conducted using vibration signal data measured from a test rig. The test rig was designed to simulate real operating conditions, collecting vibration signals under different operational states to provide real experimental data for algorithm validation. The bearing vibration dataset experimental platform is shown in Fig 18.

**Fig 19:**
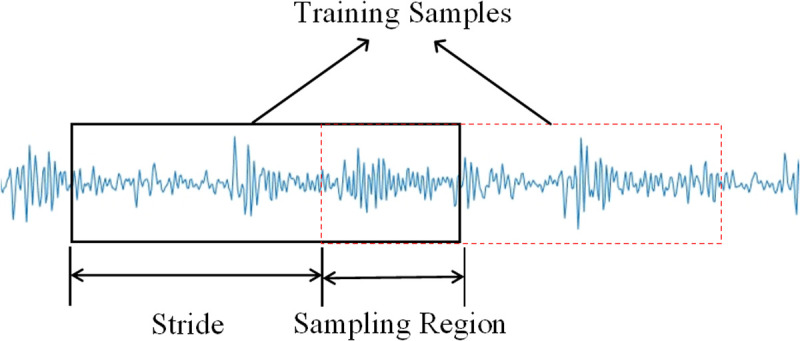
Schematic diagram of resampling. Shows the extraction of training samples from a signal using a defined sampling region and stride to generate distinct samples.

Deep learning network models often require a large number of data samples for training. In this section, an overlapping sampling method is used for data augmentation. Data augmentation not only expands the dataset but also helps the model learn different representations of the data, reducing overfitting and improving the model’s adaptability to new data. The resampling schematic diagram is shown in Fig 19.

**Fig 20:**
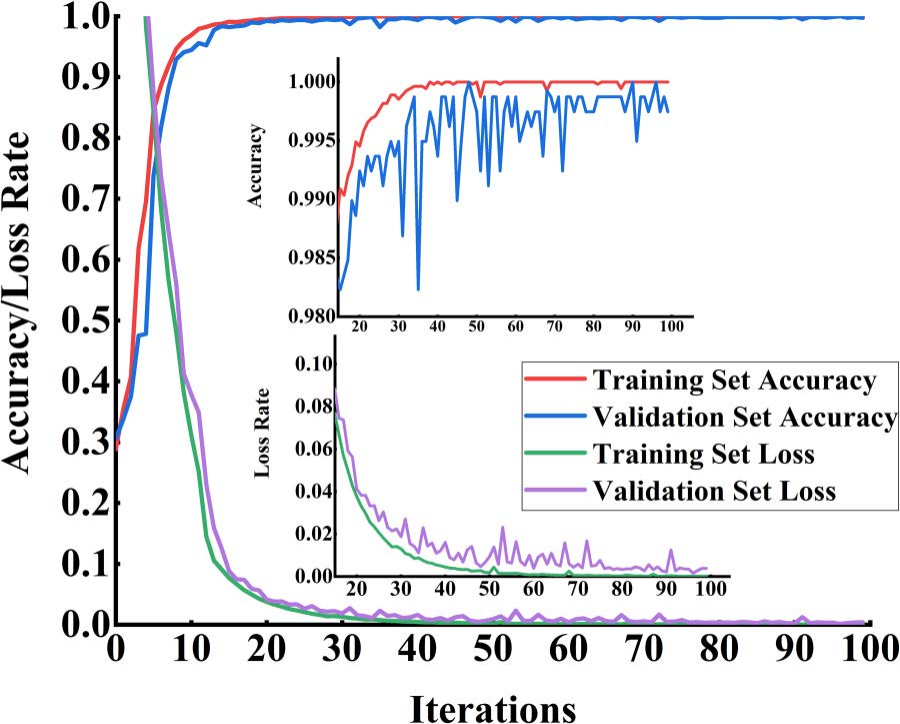
Analog signal accuracy and loss rate. Shows the changes in accuracy and loss over training iterations, reflecting the model’s learning progress and generalization performance.

The vibration signal data collected under four different conditions of rolling bearings were selected for the fault diagnosis experiment. The denoised and preprocessed vibration data were resampled and used as input samples for the fault diagnosis model. The dataset was divided into training, testing, and validation sets in a 7:2:1 ratio. The network was built using the same configuration and optimization approach as when validating the public dataset. The iterative curves of the model’s accuracy and loss rate are shown in Fig 20.

**Fig 21:**
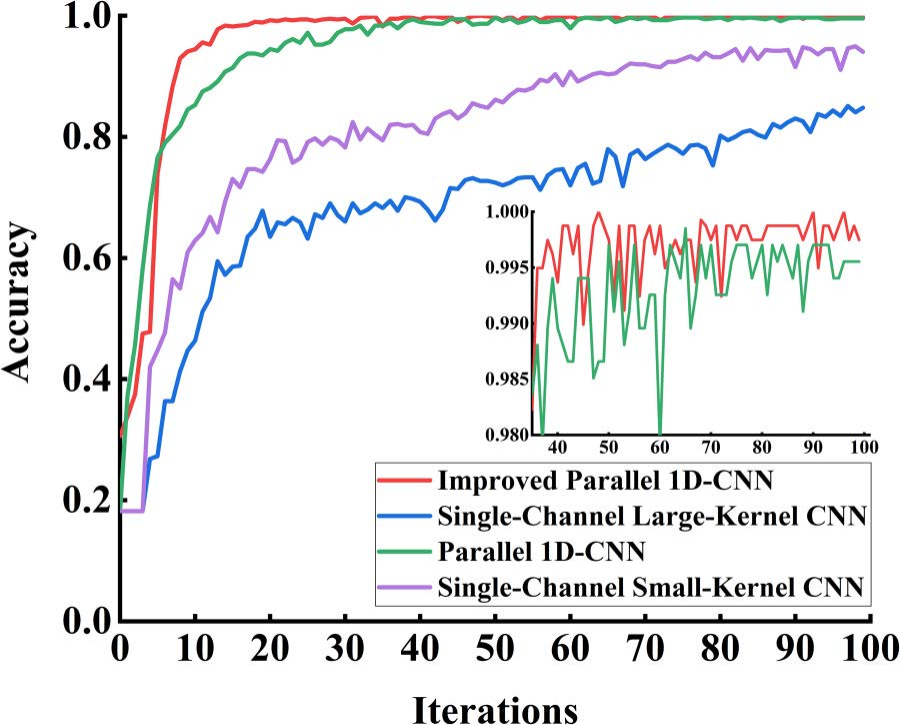
Accuracy chart of each model validation set. The improved parallel 1D-CNN model achieves higher accuracy and greater stability compared to others. The inset highlights accuracy fluctuations in later training stages.

**Fig 22:**
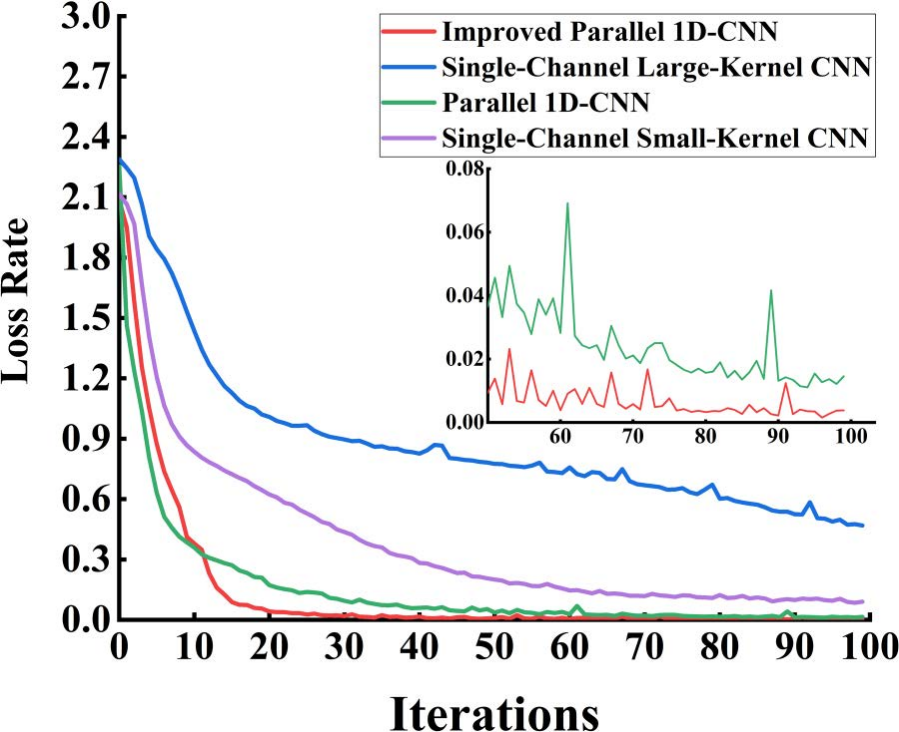
Loss rate graph of each model validation set. The improved parallel 1D-CNN model exhibits a faster loss decline and more stable performance than other models. The inset details loss fluctuations in later training stages.

As shown in Fig 19, after 25 iterations of training, both the model’s accuracy and loss rate reach a stable state, indicating that the model effectively extracts features from the data samples. Moreover, the curve exhibits a high degree of fitting, suggesting that the model does not suffer from severe overfitting or underfitting, thereby confirming the accuracy of the proposed method. To further verify the superiority of the proposed algorithm in diesel engine fault diagnosis, the same three comparison methods from the previous section were selected as benchmark models. The accuracy and loss rate of the validation set during training for each model are shown in Figs 21 and 22, respectively.

As shown in Figs 20 and 21, the curve of the single-channel 1D CNN is highly unstable, and its accuracy is lower than that of the dual-channel 1D CNN. The proposed algorithm demonstrates a faster convergence speed with minimal waveform fluctuations, indicating good stability. The test results of each model are presented in Table 4. The proposed fault diagnosis method achieves the highest diagnostic accuracy of 99.56%, which represents a 0.62% improvement over the parallel 1D CNN. Additionally, it shows a significant enhancement compared to the single-channel large-kernel CNN and the single-channel small-kernel CNN. These results confirm that the proposed algorithm has high fault identification accuracy, making it better suited for vibration signal monitoring.

**Table 4:** Accuracy and loss values of each model.

Model	Accuracy	Loss Value
**Single-Channel Large-Kernel CNN**	85.46%	0.6712
**Single-Channel Small-Kernel CNN**	92.73%	0.1943
**Parallel 1D-CNN**	98.94%	0.0225
**Improved Parallel 1D-CNN**	99.56%	0.0068

Accuracy and loss values of different fault diagnosis models evaluated on the test dataset. The improved parallel 1D-CNN achieves the highest accuracy and the lowest loss, indicating superior model performance.

#### 3.2.2 Comparative evaluation with recent state-of-the-art models.

To comprehensively assess the diagnostic performance and engineering applicability of the proposed model, we conducted a comparative study with five recently published and representative fault diagnosis approaches. These methods encompass diverse modeling paradigms, including probabilistic inference, image-based enhancement, adversarial learning, and interpretable hybrid architectures, ensuring a broad and robust benchmarking foundation.

The selected methods include:

Bayesformer: A Bayesian variational Transformer that models attention weights as stochastic distributions, enabling infinite ensemble learning and enhancing generalization capacity.

HMM-based health assessment model: Utilizes a hidden Markov process to model the stochastic degradation transitions of rolling bearings, suitable for sequential fault evolution tracking.

GADF + DSCM: Converts raw time-series signals into Gramian Angular Difference Field (GADF) images, which are then processed by a dynamic self-calibrated convolutional module (DSCM) to enhance spatial discriminability.

CWMS-GAN: A generative adversarial network that integrates continuous wavelet transform and multi-scale convolutional attention, designed to support fault diagnosis under limited-sample conditions.

QNN + BiLSTM: Combines quadratic neural networks (QNN) with bidirectional long short-term memory (BiLSTM) to enhance the nonlinear modeling and interpretability of dynamic signals.

All comparative methods were re-implemented according to the configurations reported in their respective publications. A summary of their architectural structures is as follows:

Bayesformer: Composed of three encoder blocks with Bayesian multi-head attention, a feedforward network with 256 units, and residual connections, followed by two fully connected classification layers.

HMM: Implements a three-state discrete hidden Markov model with symbol-based emissions, trained using the Baum-Welch algorithm and inferred via Viterbi decoding.

GADF + DSCM: Input signals are transformed into 64 × 64 GADF images and passed through two DSCM layers (each with 3 × 3 kernels and 32 filters), followed by global pooling and a dense output layer.

CWMS-GAN: The generator comprises three convolutional blocks with continuous wavelet transform kernels, while the discriminator consists of two convolutional layers (32 and 64 filters), an attention mechanism, and a softmax classification head.

QNN + BiLSTM: Contains two QNN layers with 128 quadratic neurons each, followed by two BiLSTM layers with 64 hidden units per direction, accompanied by dropout and softmax layers for classification.

Proposed Method: Employs a parallel dual-branch architecture. The primary branch consists of two 1D convolutional layers (32 and 64 filters), a GRU layer with 64 units, and an attention module. The auxiliary branch includes a shallow convolution layer, a pooling layer, and a fully connected layer. Features from both branches are concatenated and passed through two dense layers for final prediction.

All methods were trained on the same dataset with consistent experimental configurations wherever possible. The learning rate, batch size, number of epochs, and optimizer used for each model are summarized in Table 5.

**Table 5:** Training configurations of the compared fault diagnosis methods.

Method	Learning Rate	Batch Size	Epochs	Optimizer
**Bayesformer**	1 × 10 ⁻ ⁴	32	200	Adam with scheduler
**HMM**	N/A	N/A	50	Baum-Welch (EM)
**GADF+ DSCM**	1 × 10 ⁻ ³	64	150	SGD + Momentum
**CWMS-GAN**	5 × 10 ⁻ ⁴	16	300	Adam (β₁ = 0.5)
**QNN + BiLSTM**	1 × 10 ⁻ ⁴	64	200	RMSProp
**Improved Parallel 1D-CNN**	1 × 10 ⁻ ³	64	200	Adam (β₁ = 0.9, β₂ = 0.999)

Training configurations for the fault diagnosis methods compared in this study. The learning rate, batch size, number of epochs, and optimizer details are provided for each model. ‘N/A’ indicates that specific hyperparameters are not applicable to the corresponding method.

All input signals were standardized using Z-score normalization. Each sample consisted of a fixed length of 1200 data points. For the GADF + DSCM method, the one-dimensional signals were converted into two-dimensional Gramian Angular Difference Field (GADF) images as input. The output for all methods comprised six fault classes encoded using one-hot vectors. The classification tasks uniformly employed the cross-entropy loss function.

The experimental environment included an Intel(R) Core(TM) i7-13620H processor, an NVIDIA GeForce RTX 4060 GPU, Windows 11 operating system, Python 3.6.3 programming language, and TensorFlow-GPU 2.0.0 deep learning framework. The dataset utilized was the CWRU bearing dataset, encompassing fault categories such as normal, inner race fault, and outer race fault, among others. The data were partitioned into 70% training, 20% validation, and 10% testing subsets using stratified random sampling.

Three evaluation metrics were employed to comprehensively assess classification performance: Accuracy, representing the overall correct classification rate; Precision, indicating the model’s capability to avoid false positives; and F1-score, the harmonic mean of precision and recall, which balances fault detection and misclassification. All metrics were calculated as macro-averaged values to account for class imbalance.

Table 6 presents the performance of each method with respect to accuracy, precision, and F1-score. As shown in Table 6, the proposed method achieved accuracy, precision, and F1-score values of 99.68%, 99.65%, and 99.67%, respectively, significantly outperforming the comparative approaches. The improvements achieved by the proposed method are statistically significant (p < 0.05) according to a paired t-test over 30 repeated trials.

**Table 6:** Performance comparison of different fault diagnosis methods in terms of Accuracy, Precision, and F1-score.

Method	Accuracy (%)	Precision (%)	F1-score (%)
**Bayesformer**	98.12	97.95	98.03
**HMM**	95.74	94.82	95.28
**GADF+ DSCM**	97.86	97.10	97.44
**CWMS-GAN**	97.55	97.24	97.39
**QNN + BiLSTM**	96.43	95.61	96.02
**Improved Parallel 1D-CNN**	99.68	99.65	99.67

Comparative evaluation of fault diagnosis methods based on three key performance metrics: accuracy, precision, and F1-score. Values are expressed as percentages. Higher values indicate better diagnostic performance.

**Fig 23:**
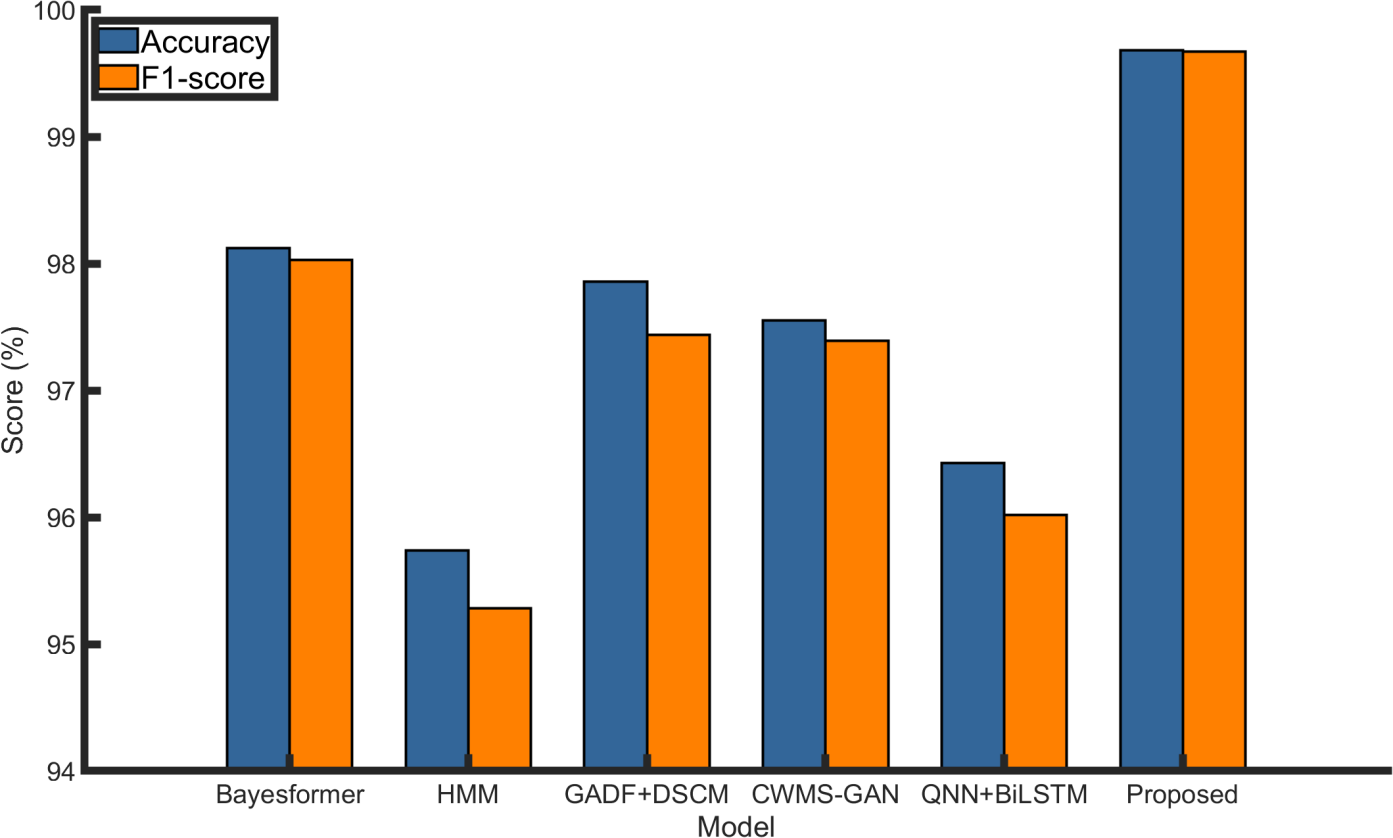
Accuracy and F1-score comparison among six advanced models. The proposed model consistently outperforms five recent methods across both metrics.

**Fig 24:**
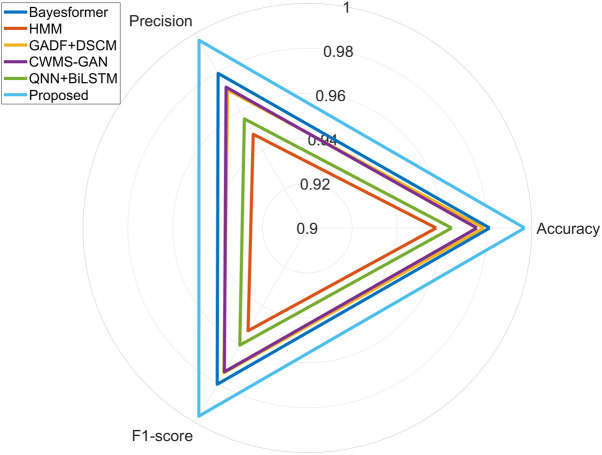
Radar chart of accuracy, precision, and F1-score. The chart highlights the superior performance of the proposed model across all three metrics.

Fig 23 illustrates a bar chart comparing the accuracy and F1-score of the six methods, clearly demonstrating the superior overall classification accuracy and stability of the proposed model. Fig 24 depicts a radar chart encompassing accuracy, precision, and F1-score, reflecting the comprehensiveness and balance of each model’s performance. Notably, the radar chart for the proposed method approximates a near-perfect circle, indicating consistent excellence across all evaluated metrics and highlighting its robustness and practical applicability in fault diagnosis. Table 7 compares the optimal application scenarios and limitations of each method, further highlighting the applicability of the proposed approach in multi-fault, noisy, and real-time contexts, while also noting its potential need for parameter tuning when deployed on lightweight devices.

**Table 7:** Comparative analysis of application scenarios and limitations of different fault diagnosis methods.

Method	Optimal Application Scenario	Limitation
**Bayesformer**	Multi-condition and few-sample environments	High model complexity and prolonged training time
**HMM**	Sequential degradation tracking	Inadequate representation of high-dimensional input features
**GADF+ DSCM**	Image-based feature learning	High preprocessing cost and sensitivity to input scale
**CWMS-GAN**	Small-sample learning	Unstable training process and risk of mode collapse
**QNN + BiLSTM**	Interpretable diagnostics	Limited capacity under noisy and complex signal conditions
**Improved Parallel 1D-CNN**	Multi-class fault diagnosis under noisy and real-time conditions	May require parameter tuning for deployment on lightweight devices

Summary of optimal application scenarios and limitations for each fault diagnosis method evaluated. The table highlights the specific conditions where each method performs best, alongside their primary drawbacks relevant to practical deployment.

The proposed model demonstrates consistently superior performance across both metrics compared with five recent methods.

The proposed method exhibits a well-balanced and dominant performance across all key indicators.

The superiority of the proposed method can be attributed to several key factors: the dual-branch architecture effectively integrates time-domain and frequency-domain features, outperforming approaches based solely on GADF or pure time-series networks; the incorporation of GRU and attention modules enhances sequence learning capabilities by focusing on critical regions, thereby avoiding underfitting common in simpler models and overfitting associated with more complex ones; the attention mechanism significantly improves discriminative power under noisy conditions, demonstrating marked advantages over HMM and CWMS-GAN in low signal-to-noise ratio environments. Additionally, although CWMS-GAN excels in small-sample scenarios and Bayesformer exhibits strong generalization ability, both entail higher computational costs. The proposed method achieves a favorable balance between complexity and performance, making it well-suited for real-time deployment in industrial settings.

## 4 Conclusion

In this paper, an improved fault diagnosis method for rolling bearings based on a parallel one-dimensional convolutional neural network (1D CNN) is proposed. By integrating dual-channel convolutional kernels, a gated recurrent unit (GRU), and an attention mechanism, the proposed approach effectively overcomes the limitations of traditional methods in processing complex signals, thereby improving the accuracy and stability of fault diagnosis. Experimental results on both publicly available datasets and real vibration signals demonstrate that the proposed method outperforms existing approaches in terms of diagnostic performance. These findings confirm the effectiveness of the proposed improvements in enhancing feature extraction and classification accuracy. However, despite the notable advantages, certain limitations remain. The computational efficiency of the model requires further optimization, particularly when handling large-scale datasets. Future work will focus on refining the model structure, enhancing its generalization ability, and improving computational efficiency. Additionally, further investigations will be conducted to explore its practical applications in complex industrial environments.
